# The risk of mpox importation and subsequent outbreak potential in Chinese mainland: a retrospective statistical modelling study

**DOI:** 10.1186/s40249-024-01189-1

**Published:** 2024-02-29

**Authors:** Xiaowei Deng, Yuyang Tian, Junyi Zou, Juan Yang, Kaiyuan Sun, Hongjie Yu

**Affiliations:** 1 School of Public Health, Key Laboratory of Public Health Safety, Ministry of Education, Fudan University, Shanghai, 200032 China; 2 Division of International Epidemiology and Population Studies, Fogarty International Center, National Institutes of Health, Bethesda, MD USA

**Keywords:** Mpox (Monkeypox), Importation risk, International air travel, Statistical modelling, Men who have sex with men

## Abstract

**Background:**

The 2022–2023 mpox (monkeypox) outbreak has spread rapidly across multiple countries in the non-endemic region, mainly among men who have sex with men (MSM). In this study, we aimed to evaluate mpox’s importation risk, border screening effectiveness and the risk of local outbreak in Chinese mainland.

**Methods:**

We estimated the risk of mpox importation in Chinese mainland from April 14 to September 11, 2022 using the number of reported mpox cases during this multi-country outbreak from Global.health and the international air-travel data from Official Aviation Guide. We constructed a probabilistic model to simulate the effectiveness of a border screening scenario during the mpox outbreak and a hypothetical scenario with less stringent quarantine requirement. And we further evaluated the mpox outbreak potential given that undetected mpox infections were introduced into men who have sex with men, considering different transmissibility, population immunity and population activity.

**Results:**

We found that the reduced international air-travel volume and stringent border entry policy decreased about 94% and 69% mpox importations respectively. Under the quarantine policy, 15–19% of imported infections would remain undetected. Once a case of mpox is introduced into active MSM population with almost no population immunity, the risk of triggering local transmission is estimated at 42%, and would rise to > 95% with over six cases.

**Conclusions:**

Our study demonstrates that the reduced international air-travel volume and stringent border entry policy during the COVID-19 pandemic reduced mpox importations prominently. However, the risk could be substantially higher with the recovery of air-travel volume to pre-pandemic level. Mpox could emerge as a public health threat for Chinese mainland given its large MSM community.

**Graphical Abstract:**

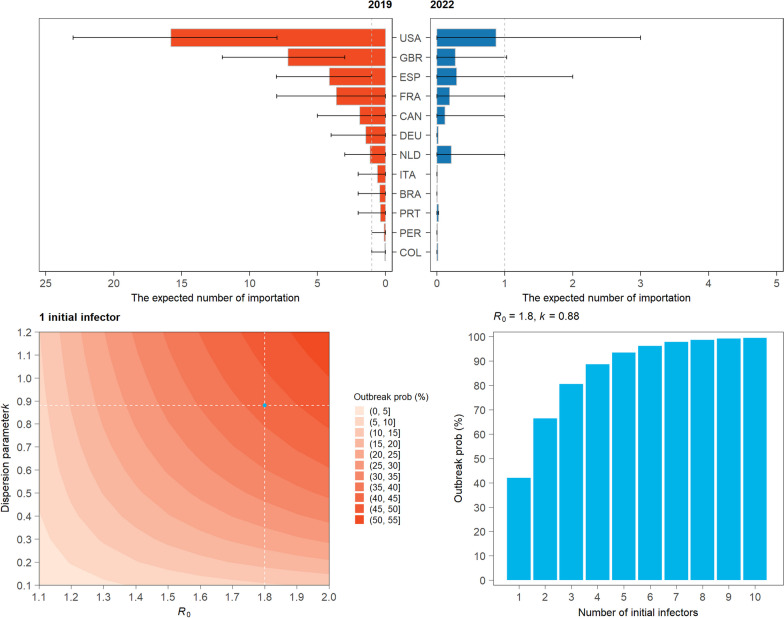

**Supplementary Information:**

The online version contains supplementary material available at 10.1186/s40249-024-01189-1.

## Background

Since the first human infection was detected in Central Africa in 1970 [1], the monkeypox caused by monkeypox virus has been recognized as a viral zoonosis with endemic regions primarily concentrated in west and central African countries [2, 3]. The monkeypox virus, a member of the *Orthopoxvirus* genus in the *Poxviridae* family, is closely related to the widely known variola virus (which causes smallpox) [4]. In the last two decades, the global incidence of monkeypox cases (here after, monkeypox is denoted as mpox according to World Health Organization (WHO) reference) has been on the rise in endemic regions, with an increase in the median age among confirmed and probable mpox cases over time [5]. Before the 2022–2023 multi-country mpox outbreak, imported mpox infections in humans have been reported in the USA [6–9], UK [10], Israel [11], and Singapore [12].

After the confirmation of the mpox case in England on May 7, 2022, the outbreak has rapidly expanded spatially to other countries in Europe, the Americas, and Western Pacific region [13]. It is the first time that mpox cases have been reported in both endemic and non-endemic regions with broad geographical coverage. This multi-country mpox outbreak also reveals different epidemiological and clinical characters from previous outbreaks [7, 14–18] (Additional file 1: Table S1). In particular, sustained human-to-human transmission has been observed, involving predominately men who have sex with men (MSM) [19], which made mpox as a sexually transmitted disease (STD) [20]. On July 23, 2022, the WHO declared the multi-country outbreak of mpox as a Public Health Emergency of International Concern (PHEIC) due to its unprecedent rapid spread in the non-endemic regions and disparate global dissemination [21, 22], and on May 11, 2023, the WHO canceled the PHEIC declaration. Since January, 2023, countries/regions in East Asia have started experiencing local mpox outbreaks that were not seen during the height of mpox outbreak in 2022 [23]. As of June 15, 2023, 181 confirmed cases have reported by Japan and 102 cases by the Republic of Korea [24, 25]. Taiwan province in China reported over 50 local mpox cases in May 2023. It is crucial to evaluate the mpox outbreak potential in Chinese mainland, due to its large pool of susceptible population at risk of mpox and the fundamental change in the geographical range of mpox circulation.

In this study, we used mathematical models to evaluate: (1) the importation risk by international air travel from countries with ongoing outbreaks; (2) the effectiveness of two sets of border screening strategies considering Chinese mainland’s pre- and peri-pandemic (hypothetical) border entry and quarantine policies; (3) the risk of undetected mpox importations to trigger a local outbreak in Chinese mainland across a range of plausible mpox transmissibility and contact patterns among the MSM community.

## Methods

We constructed a probabilistic model using reported mpox cases data and international air-travel data to estimate the according importation risk in Chinese mainland between April 14 and September 11, 2022. We also simulated effectiveness of two border screening strategies, and estimated the local outbreak probability considering different importation cases and parameters.

### Simulating the international dissemination of the 2022–2023 multi-country mpox outbreak and evaluating Chinese mainland’s importation risk

To quantify the international importation risk of mpox from a source location $i$ (regions with ongoing mpox transmission) to a destination location $j$, we need to know the outbreak size in the source location $i$ and the connectivity between the origin (location $i$) and destination (location $j$) by air-travel. We assumed that travel-associated mpox cases that contribute to the international spread mostly reside in major metropolitan areas with international airport transportation hubs. Hence, we considered a total of 39,114 mpox cases which occurred in 39 metropolitan areas with major international airports in the top 12 countries affected by mpox (Additional file 1: Tables S2 and S3) and evaluated their risk of exportations to Chinese mainland. Specifically, we obtained the time series of mpox incidence acquired outside Chinese mainland at the national level from Global.health [26]. We removed all travel-related cases from our analysis and assumed the rest of cases reside in the location of reporting and acquired infection in the location of reporting. We then decomposed the national-level time series into metropolitan-level proportional to the relative outbreak size of a metropolitan area with respect to its national total. For each mpox infection at the source location $i$, we assumed that the infected individual could only make international air travel during the incubation period of $\tau$ days (i.e., prior to symptom onset), which we drew from a lognormal distribution with an average of 8.94 days and standard deviation of 4.19 (Additional file 1: Table S4). For each case, we assumed there is a delay of $\sigma$ days between symptom onset and the date of reporting, drawing from a zero-inflated negative binomial distribution with an average of 6.48 days and a standard deviation of 28.2 days (Additional file 1: Figure S1, Section S4). Consequently, if an mpox case is reported on date $t$, this infected individual could potentially travel internationally between date $t-\sigma$ and date $t-\sigma -\tau$, i.e., during his/her incubation period.

We then estimated the international air-travel probability using the origin–destination air-travel data provided by OAG. The pandemic greatly influenced global air travel. We considered a pre-pandemic (upper-bound) scenario using the air-travel data of year 2019 and a peri-pandemic (lower-bound) scenario using the air-travel data of year 2022. For each air-travel scenario, if we denote the total air-travel volume at month $m$ from location $i$ to location $j$ as ${\Phi }_{ij}^{m}$ (provided by OAG), the per-capita rate of international air travel ${\phi }_{ij}$ can be expressed as ${\phi }_{ij}=\frac{{\Phi }_{ij}^{m}}{{N}_{i}{D}_{m}}$, where ${N}_{i}$ is the total population in location $i$ and ${D}_{m}$ is the number of days in month $m$. We then iterated through all mpox cases and simulated each of their potential air-travel trajectory during their incubation period $\tau$: starting from the beginning of the incubation period $t-\sigma -\tau$, for each day forward $t{\prime}$ till the end of the incubation period $t-\sigma$, whether the individual would travel on date $t{\prime}$ is simulated as a Bernoulli process with the probability of travel ${p}_{i}\left(t{\prime}\right)$ from location $i$ is given by ${p}_{i}={\sum }_{j}{\phi }_{ij}\left(t{\prime}\right)$. Given that the individual would travel on $t{\prime}$, then the destination $j$ of the international travel would be decided based on a generalized Bernoulli distribution with the conditional probability of traveling to a specific location $j$ given by $\frac{{\phi }_{ij}\left(t{\prime}\right)}{{\sum }_{j}{\phi }_{ij}\left(t{\prime}\right)}$ and the simulation for this infected individual ends. The simulation of the infected individual would also end if by the end of his/her incubation period $t-\sigma$, the infected individual does not travel, and this individual would remain as a local mpox case in location $i$. Once we iterate through all mpox infections globally, we could sum up the cumulative number of mpox importations from location $i$ to location $j$ up to time point $t$, denoted as ${m}_{ij}\left(t\right)$. We repeated the entire simulation for a total of 200 times and obtained the distribution of ${m}_{ij}\left(t\right)$, denoted as $P\left({m}_{ij}\left(t\right)\right)$ to capture the stochastic nature of the modelled process (Additional file 1: Figure S2). We could estimate the mean and 95% confidence interval (0.025 and 0.975 quantiles) of ${m}_{ij}\left(t\right)$ based on $P\left({m}_{ij}\left(t\right)\right)$. To ensure the validity of our model, we performed a validation analysis of the mpox importations among countries where consecutive reports of mpox cases with international travel history were available, with abovementioned pre-pandemic and peri-pandemic air-travel volume setting the upper bound and lower bound of the importation risks, respectively (Additional file 1: Figure S3).

### Quantifying the effectiveness of border screening against travel-associated mpox cases

To quantify the effectiveness of border screening policies, we first considered a scenario (denoted as Scenario 1 hereafter) that is in compliance with Chinese mainland’s current pandemic quarantine policy for border entry: according to the recently published (July 1, 2022) guidelines to mpox prevention and control by National Health Commission of the People’s Republic of China, all international travelers entering Chinese mainland, especially for those who had travel history to regions with an on-going mpox epidemic within 21 days of border entry, should be screened for mpox during their mandatory quarantine as a part of Chinese mainland’s pandemic response [27]. During the quarantine, individuals are also screened for mpox related symptoms including fever and rash. Symptomatic individuals with travel history from outbreak countries, close contact with confirmed cases, or contact with infected animals would be defined as suspected cases (Additional file 1: Table S5). Once detected, they would be reported to the local Centers for Disease Control and Prevention (CDC) and will be transferred to designated hospitals for a 21-day medical observation, and PCR test for lesion samples/swabs/blood will be conducted. Considering the variation of pandemic mandatory quarantine durations (ranging from 7 to 21 days) as well as the uncertainty on the proportion of people with mpox infections complying with self-reporting, we used a mathematical model to evaluate the proportion of imported mpox infections detected post entry screening (Additional file 1: Section S8). We also considered another hypothetical scenario (denoted as Scenario 2 hereafter) of plausible mpox entry screening in the absence of Chinese mainland’s pandemic quarantine policy for international travelers (a pre-pandemic scenario). In this scenario, international travelers with a self-reported mpox epidemiological link need to undergo medical observation for mpox-related symptoms (e.g., fever, rash, lymphadenopathy) during which they abstain from sexual activity, in addition to other precautionary measures [28]. Upon symptom onset, a suspected mpox case needs to self-isolate and seek testing. We also evaluated the effectiveness of this travel screening policy (Additional file 1: Section S8).

### Estimating local outbreak probability

Following Hartfield et al.’s analytical solution of outbreak probability as a function of the basic reproduction number ${R}_{0}$ and overdispersion parameter $k$ [29] (Additional file 1: Section S9), we explored the range of mpox outbreak probability with one initial infector introduced into the high-risk population in Chinese mainland, with ${R}_{0}$ ranging from 1.1 to 2.0 and dispersion parameter $k$ ranging from 0.1 to 1.2 (Fig. 4a). Our best guess for ${R}_{0}$ for the 2022 mpox outbreak is 1.8, according to a recent study conducted by Kwok et al. [30], and we considered a plausible dispersion parameter $k$ of 0.88 through fitting the negative binomial distribution for the number of sexual partners among MSM in China [31, 32] (Additional file 1: Figure S4). For ${R}_{0}$ = 1.8 and $k$ = 0.88, we explored how the outbreak probability would change with the number of imported mpox cases missed by the border screening process (Fig. 4b). To understand the effect of emergency vaccination on preventing the outbreak probability, we used ${R}_{t}$ to stand for the protection provided by emergency vaccination. The relationship can be illustrated as ${R}_{t}={R}_{0}\times S$, in which *S* stands for the proportion of population susceptible to mpox. We ignored the complexity of the different VE of one/two dose mpox vaccination in the US [33, 34]. We simply assumed 20% high risk population get effectively vaccinated, hence the ${R}_{t}$ = 1.46.

### Exploring the contribution of air travel and active MSM population size on outbreak in China

During June to November 2023, China has experienced a certain outbreak. We extracted monthly provincial reported number of mpox cases, and implemented a generalized linear model to testify the contribution of air-travel volume and active MSM population size at the province level estimated by Hu et al. [35]. We modeled with the permutation of these two metrics and the interaction.

All analysis was done in R (version 4.1.1; R Foundation for Statistical Computing, Vienna, Austria; https://www.r-project.org/).

## Results

### Chinese mainland’s mpox importation risk

**Fig. 1:**
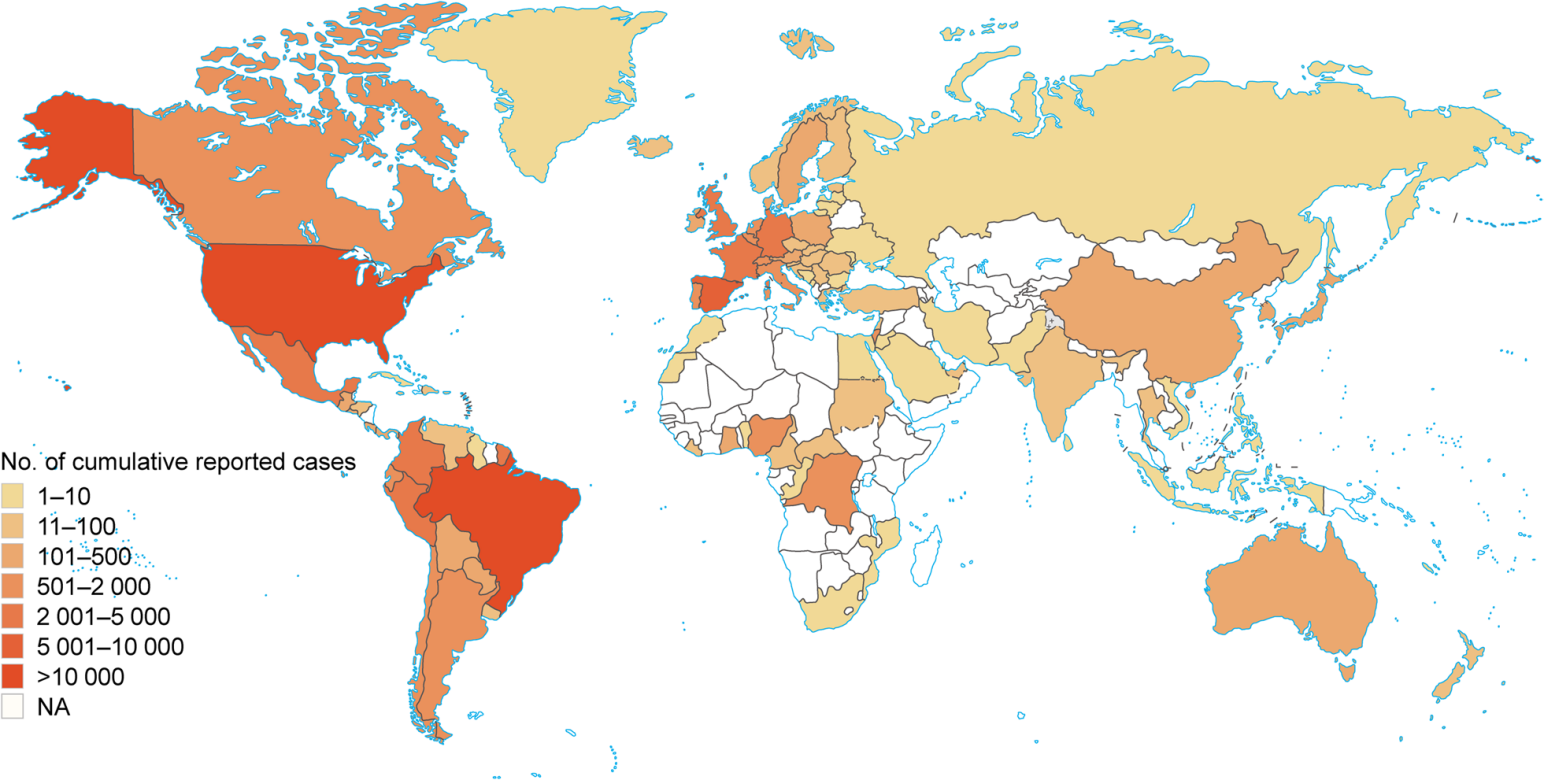
Spatial distribution of mpox cases. Spatial distribution of confirmed mpox cases from January 1, 2022 to June 13, 2023. Shapes in color of ivory indicate that data in countries/regions is not available. Map approval No.: GS (2024) 0409

According to the WHO situation report [36], from January 1, 2022 to June 13, 2023, a total of 87,979 confirmed mpox cases were reported among 111 countries and regions, with the majority of cases reported in the American (59,449) and European (25,910) regions, accounting for 67.6% and 29.5% of the global cases (Fig. 1).

**Fig. 2:**
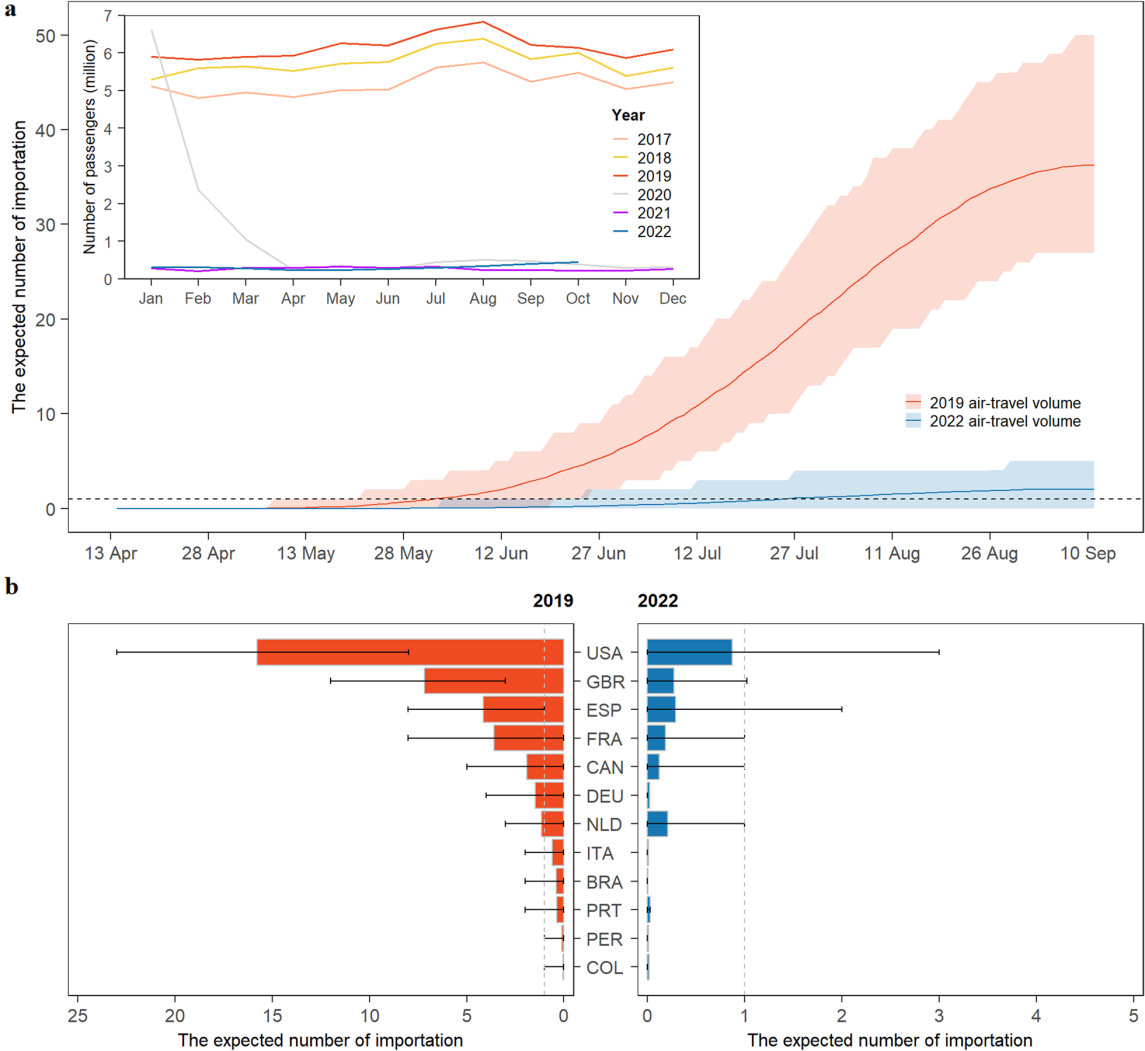
The air-travel volume and the expected number of mpox importations in China. **a** The expected cumulative number of mpox importations from May to September 2022 with air-travel volume in 2019 and 2022. **b** The expected number of mpox case importation from other countries/regions with air-travel volume in 2019 and 2022. In panel **a**, the inset indicates the actual monthly air-travel volume to Chinese mainland from 2017 to 2022. The red and blue curves in the main panel indicate the expected number of mpox importations in the Chinese mainland. In panel **b**, the three letter texts are ISO Alpha-3 codes, BRA denotes Brazil, CAN denotes Canada, COL denotes Colombia, DEU denotes Germany, ESP denotes Spain, FRA denotes France, GBR denotes United Kingdom, ITA denotes Italy, NLD denotes Netherlands, PER denotes Peru, PRT denotes Portugal, USA denotes United States of America (Additional file 1: Table S8)

According to the air-travel data from OAG [37], we found the monthly number of arrivals in China was on average 5.69 million during 2017–2019. However, the volume drastically decreased to 0.6 million starting in January 2020, approximately one-tenth of the pre-pandemic (2017–2019) air-travel volume (Fig. 2a, inset). Assuming a hypothetical scenario that the year 2022 has the same air-travel volume as in 2019 (pre-pandemic), we estimated that the expected number of Chinese mainland’s mpox importations would have risen above one on June 2, 2022 and reached 36 [95% confidence interval (*CI*) 27–50] on September 11, 2022. In contrast, according to the actual international air-travel volume during 2022 (peri-pandemic), we estimated that the expected cumulative importations would only reach two (95% *CI* 0–5) by September 11, 2022. In Fig. 2b, we presented Chinese mainland’s top 12 source countries for mpox importations based on the travel volume of year 2019 and 2022, respectively. Under the air-travel volume of 2019, Chinese mainland would expect (on average) more than one mpox infection importation from epidemic countries including the United States (16, 95% *CI* 8–23), the United Kingdom (7, 95% *CI* 3–12), Spain (4, 95% *CI* 1–8), France (4, 95% *CI* 0–8), Canada (2, 95% *CI* 0–5), Germany (1, 95% *CI* 0–4) and Netherlands (1, 95% *CI* 0–3). In contrast, under the air-travel volume of 2022, we estimated that no single source country would contribute to more than one expected mpox importation to Chinese mainland, with the highest-ranking country (the United States) contributing to about one mpox importation (95% *CI* 0–3).

### Effectiveness of border screening policy against mpox importation

**Fig. 3:**
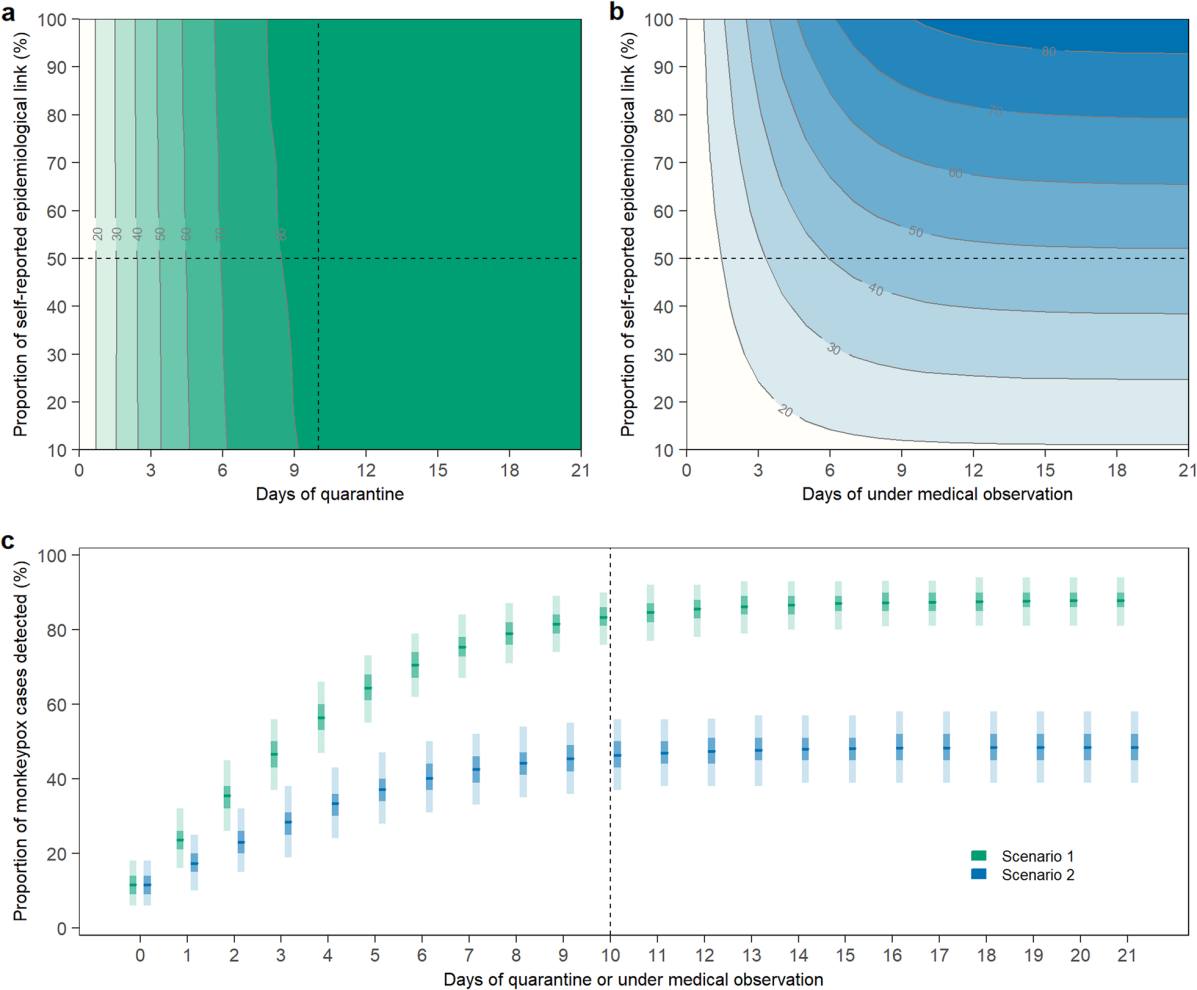
Effectiveness of mpox border screening policies under two assumed scenarios. **a** Number of total detected mpox cases under Scenario 1 (corresponding to China’s pandemic quarantine policy), with proportion of self-reported epidemiological links ranging from 10 to 100%, and days of quarantine ranging from 0 to 21. **b** Number of total detected mpox cases under Scenario 2 (a pre-pandemic scenario), with proportion of self-reported epidemiological links ranging from 10 to 100%, and days of medical observation ranging from 0 to 21. **c** Number of total detected mpox cases with 50% self-reported epidemiological links with mpox cases under days of quarantine or medical observation from 0 to 21. In panel c, the horizonal lines indicate the mean, with dark and light shades indicating 50% *CI*, and 95% *CI*

We first examined the effectiveness of an mpox border screening policy (measured as the probability of mpox infection detection among all mpox importations) under Chinese mainland’s pandemic quarantine policy for international travelers (Scenario 1). Specifically, we found that under Scenario 1, the effectiveness of border screening against mpox is insensitive to the self-reporting rate of an mpox epidemiological links, as all international travelers were required to quarantine due to existing pandemic policies. On the other hand, the detection rate of mpox importations increases with the quarantine duration (Fig. 3a). Chinese mainland’s pandemic policy requiring a quarantine duration of 10 days (policy as of June 28, 2022 [38]) would lead to high effectiveness of mpox screening with detection rate reaching > 80% based on our model’s estimate, irrespective of the self-reporting rate of epidemiological links. However, universal border entry quarantine as a stand-alone policy for mpox, in the absence of pandemic-related policy, is impractical and unwarranted due to mpox’s much slower spread compared to COVID-19 and the lack of evidence of pre-symptomatic transmission [28]. We thus considered a scenario of a border screening policy similar to the contact tracing guidance by the European Center for Disease Prevention and Control (ECDC) [28]. In this scenario, both the self-reporting rate of epidemiological links and the duration of the medical observation influence the screening effectiveness: the proportion of imported mpox infections who self-report an epidemiological links sets the upper bound of the mpox border screening effectiveness, as infected individuals who do not report/are unaware of mpox exposure would not undergo medical observation and thus could not be promptly detected (Fig. 3b). Among exposed individuals undergoing medical observation, increasing the duration of medical observation would increase the detection rate of border screening but the increase would start plateauing with only marginal benefit once the duration reached beyond 10 days. In Fig. 3c, we compared the overall effectiveness of Scenario 1 against Scenario 2 across a range of quarantine/medical observation durations, conditional on the same proportion of self-reporting epidemiological link (50%). We found that the more stringent Scenario 1 always outperforms Scenario 2 across different quarantine/medical observation durations. At the quarantine/medical observation duration of 10 days, Scenario 1 on average detects 83.3% of imported mpox cases while Scenario 2 could only detect 46.3%. Longer quarantine/medical observation duration does not significantly improve the border screening effectiveness for either of the two scenarios.

### Local mpox outbreak probability

**Fig. 4:**
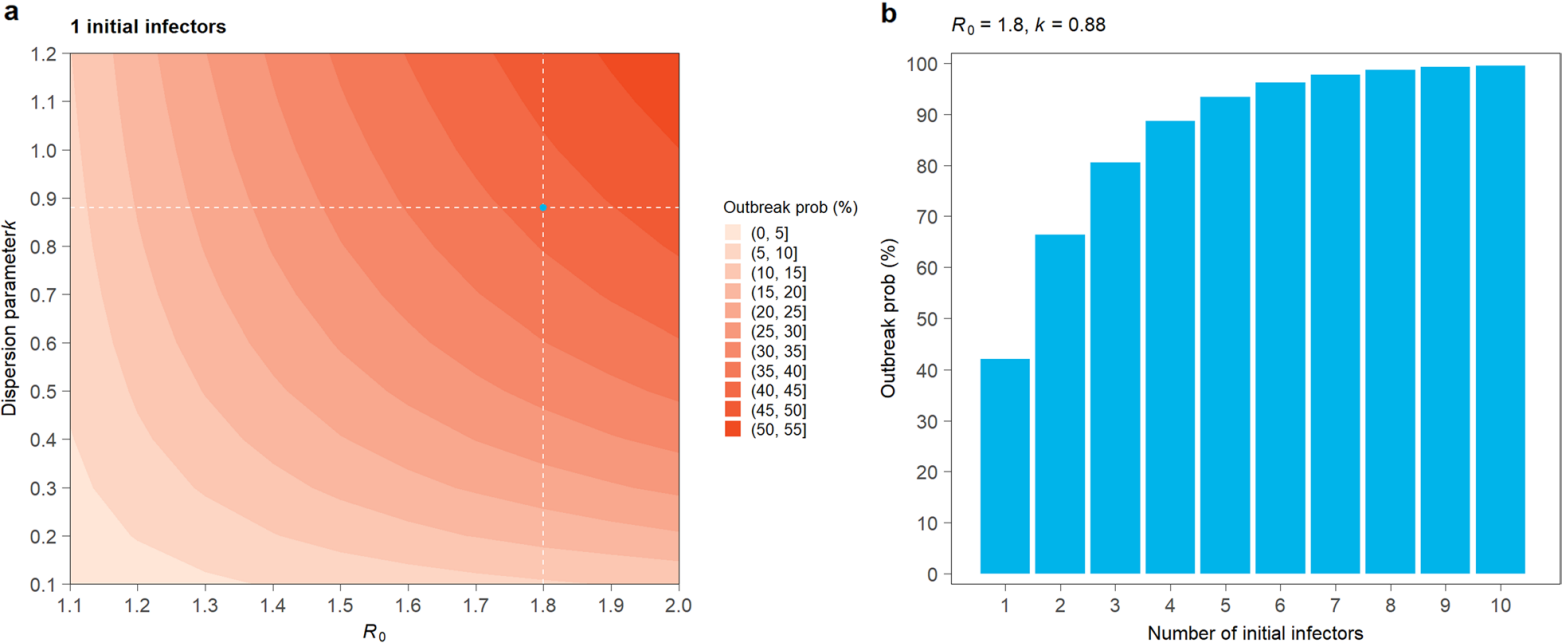
Estimated outbreak probability. **a** the outbreak probability of one mpox case introduced into the MSM community in Chinese mainland, with *R*_0_ ranges from 1.1 to 2, and dispersion parameter *k* ranges from 0.1 to 1.2. In panel a, the dashed lines and dot refer to *R*_0_ = 1.8 and *k* = 0.88, a plausible guess of mpox transmissibility in Chinese mainland. **b** the outbreak probability as a function of the number of seeding infectors ranges from 1 to 10, with *R*_0_ = 1.8 and *k* = 0.88

Next, we used a mathematical model to evaluate the potential of undetected mpox infections triggering a local mpox outbreak. The outbreak potential of an undetected mpox importation is influenced by the overall transmissibility of the mpox virus (measured as the basic reproduction number ${R}_{0}$) and the transmission heterogeneity (measured as the overdispersion parameter $k$, Additional file 1: Section S9) [29]. In Fig. 4a, we demonstrated the outbreak probability of a single undetected mpox infection in the high-risk population as a function of ${R}_{0}$ and $k$: both the increasing of ${R}_{0}$ (increasing transmissibility) and $k$ (decreasing transmission heterogeneity) would increase the outbreak probability. Considering the recent estimation of ${R}_{0}$ = 1.8 for mpox outbreaks in several European countries, the outbreak probability caused by one undetected mpox case was estimated at 42.1% if the overdispersion parameter $k$ was equal to 0.88, based on sexual contact network among MSM in Chinese mainland [31, 32]. The outbreak probability drastically goes up with the number of undetected mpox importations, reaching to above 95% with six or more importations. When taking into account of emergency vaccination, the reproduction number is reduced to 1.46 and the outbreak probability accordingly reduces to 29.4% with only one imported mpox case, which is lower than the probability of 70% when ${R}_{0}$ = 1.8.

### Contribution of air travel and active MSM population size

Furthermore, we explored the correlation between the confirmed mpox cases and active MSM population size. The result shows high correlation with *R* = 0.72 (*P* < 0.05) when we removed Beijing and Guangdong province (both are major international air-traffic hubs for Chinese mainland) presenting as outliers (Additional file 1: Figure S5). We further performed a regression model including both mpox cases, active MSM population size and air-travel volume as covariate, with and without removing Beijing and Guangdong (Additional file 1: Section S11). When included both two factors and their interaction and removed Beijing and Guangdong, the result of the regression showed high coefficient of determination (*R*^2^ = 0.84), which is lower with removing the interaction (*R*^2^ = 0.62).

## Discussion

Since the global eradication of smallpox in the 1980s and subsequent halt of the smallpox vaccination program, it has been suggested that the mpox virus could emerge as the next *Orthopoxvirus* of epidemic potential in humans, taking up the ecological niche vacated by smallpox in the human population [39]. In recent years, increasing incidence and shifting demographics towards older populations and males in the endemic region were warning signs of declining of mpox population immunity due to the cessation of smallpox vaccination in the 1980s and population turnover. Following the first confirmed mpox case reported by the United Kingdom in early May 2022, the multi-country mpox outbreak had infected about 90,000 individuals in non-endemic regions as of September 12, 2023, with countries in Europe and the Americas being impacted most heavily. After confirming mpox’s local transmission, the UK and other European countries subsequently adopted contact tracing and case isolation to control their outbreaks [40, 41]. Following the PHEIC declaration of the outbreak by the WHO, the US government declared a public health emergency for the local mpox outbreak and adopted measures (case identification, contact tracing, vaccination) to suppress local transmission. When faced with the importation risk of mpox, only a few countries have strengthened their border screening to prevent case importation [42–47].

On September 16, 2022, Chinese mainland recorded its first imported mpox case in Chongqing. The case had a travel history to Germany and Spain [48] and remained the only observed mpox importation as of June 5, 2023. This observed low risk of mpox importation is in line with our model’s projections based on 2022 air-travel volume. By examining the OAG’s data through October 2022, Chinese mainland’s air travel has yet to show significant recovery from that of 2022 (Fig. 2a). Furthermore, as a byproduct of the universal quarantine policy for border entry due to the pandemic, our analysis suggests high border screening effectiveness for mpox if medical observation for mpox were implemented during the mandatory quarantine. Collectively, the impact of pandemic on air-travel reduction and a stringent quarantine policy have led to a low risk of mpox importation in Chinese mainland. However, under conditions where air-travel volume is gradually rebounding to pre-pandemic levels, we estimate that the importation risk is substantially higher, reaching 36 (95% *CI* 25–47) mpox importations under the current outbreak (Fig. 2a).

A distinctive feature of the 2022 mpox outbreak is that it affects predominantly the population in the MSM community with sustained human-to-human transmission outside the endemic region [13]. Modeling analysis of the UK’s sexual contact network of the MSM community suggested the heavy-tailed nature of network topology could result in the basic reproduction number of mpox larger than the epidemic threshold of one among the group of individuals who have disproportionately large numbers of partners [49]. We have looked at the distribution of sexual partners among MSM in China in 2010 based on the study by Zhang et al. [31, 32] (Additional file 1: Figure S4); it is highly heterogeneous, with 4.9% of individuals having more than 10 sexual partners over a period of one year. Thus, it is plausible to expect that once mpox infection is introduced into the MSM community in Chinese mainland, the mpox virus could sustain human-to-human transmission and cause substantial disease burden in the high-risk population. Assuming the mpox’s basic reproduction number in Chinese mainland is similar to that in Europe and the United States [30], we estimated that the probability of triggering a local mpox outbreak is substantial and rapidly goes up with the number of mpox introductions (Fig. 4). Noticeably, the highly heterogeneous sextual contact network structure could significantly reduce the outbreak size when comparing to a homogeneous mixing model [49], and ${R}_{0}$ is not a good predictor of outbreak size. We only use ${R}_{0}$ alone with dispersion parameter *k* to evaluate the risk of triggering local outbreak. We do not use ${R}_{0}$ to evaluate the final outbreak size. A recent study suggests that the size of the MSM community in Chinese mainland is approximately eight million [35], roughly twice the size of that in the US [50]. With the changing epidemiology and population immunity of mpox as well as the geographical expansion to non-endemic regions, mpox has emerged as a public health threat for Chinese mainland’s MSM community in the foreseeable future.

Although the global mpox epidemic trend has been on the decline since August, 2022, and the WHO declared that the mpox epidemic no longer constituted a PHEIC, mpox is still circulating with low intensity in non-endemic countries/regions recently [22]. Several Asian countries including Japan, Thailand, etc. have reported imported and local mpox cases since May, 2023 [23, 51]. Taiwan Province in China reported over 50 local mpox cases in May, 2023. From June to August, 2023, 26 provincial-level administrative divisions in Chinese mainland reported 1098 confirmed mpox cases, suggesting likely wide-spread mpox local outbreaks in Chinese mainland. This is in agreement with our modeling assessment of high mpox outbreak probability in Chinese mainland, conditional on introduction of travel-related mpox cases (Fig. 2). From our exploration on the relationship between the local number of mpox cases with air-travel volume and active MSM population size at the province level (Additional file 1: Section S10), we found that both the active MSM population size and air-travel volume are positively correlated with the number of reported mpox cases, especially for the interaction of them.

Our study has several limitations. First, due to the data inaccessibility, we assumed that the air-travel behavior of the population in the MSM community is the same as that of the general population, which may not be true and could variate between different demographic profiles. Also, the air-travel data we are using is not publicly accessible. However, international air-travel data can also be approximated by using a model applied to international trade data [52] which is publicly available. This provides a freely accessible alternative to the commercial air-travel data [37]. Second, we assumed there is no under-reporting of mpox infections, which may lead to an underestimation of the importation risk in Chinese mainland. However, considering the high symptomatic rate of mpox infection as well as distinct symptom presentations including fever and rash, we expect our estimation and our conclusion remain robust. Lastly, data on MSM sexual contact networks are limited in Chinese mainland. In particular our MSM sexual contact network is based on data pre-pandemic and during the pandemic, the sexual behavioral could have changed substantially. Better characterization of the behavioral aspect of the high-risk population could further improve our evaluation of mpox outbreak potential and define effective preventive and mitigation public health policies against mpox.

## Conclusions

Using pre- and peri-pandemic air-travel data and spatiotemporally resolved mpox case reporting, our study demonstrates that the reduced international air-travel volume and stringent border entry policy during the COVID-19 pandemic reduced mpox importations prominently. However, the risk could be substantially higher with the recovery of air-travel volume to pre-pandemic level. Mpox could emerged as a public health threat for Chinese mainland given its large MSM community.

### Supplementary Information

**Additional file 1: Table S1.** Summary of epidemiological and clinical features. **Table S2.** Summary of case number and population of each metropolitan areas among top 12 countries affected by mpox. **Table S3.** Summary of international airports for each metropolitan area. **Table S4.** Fitting information for duration of rash. **Figure S1.** Distribution of the interval from onset to report. (a) The probability density function. (b) The cumulative distribution function. **Figure S2.** The diagram of simulating the international dissemination of the 2022 multi-country mpox outbreak and evaluating Chinese mainland’s importation risk. In the left panel, we firstly removed all possible imported cases in location *i* and randomly generated their infectious period according to incubation period. In the right panel, we randomly determine the travel date from their infectiousness period and generate the destination *j* according to the travel volume. We iterated the entire simulation for 200 times and obtained the distribution of the mpox importations number from location *i* to location *j* up to time point *t*. **Figure S3.** Validation of the importing risk estimation for 24 countries reporting importation cases more than three times. In each panel, the red ribbon indicates the estimation based on air-travel volumn of 2019 (upper bound estimation) and the blue one indicates that of 2022 (lower bound estimation). Purple dots indicate the observed importations. **Table S5.** Case Definitions of mpox in Chinese mainland. **Table S6.** Summary of diagnostic accuracy of confirmation test. **Figure S4.** Distribution of the number of sexual partners within 1 year among MSM community in Chinese mainland. (a) The cumulative distribution in Chinese mainland. (b) The probability mass distribution of the number of MSM sexual partners in Chinese mainland. In panel a, dots in color of yellow indicated raw data extracted from literature (45, 46). **Figure S5.** Correlation between the confirmed mpox cases number and active MSM population size of 29 provinces in Chinese mainland. The orange ribbon indicates the estimation based on the active MSM population size(47). The blue dots indicate different provinces in Chinese mainland and the two red dots present outliers in the analysis. **Table S7.** *R*^2^ for different regression combination. **Table S8.** Country/region ISO Alpha-3 code and the according UN name.

## Data Availability

All code and publicly accessible data used during the study are available at https://github.com/DXW-sola1015/Mpox-importation-risk. International air-travel data is obtained from OAG and can not be made public.

## Abbreviations

WHO: World Health Organization
MSM: Men who have sex with men
STD: Sexually transmitted disease
PHEIC: Public Health Emergency of International Concern
OAG: Official aviation guide
ECDC: European Center for Disease Prevention and Control
CDC: Centers for Disease Control and Prevention
