# Supplementary material for: The risk of mpox importation and subsequent outbreak potential in Chinese mainland: a retrospective statistical modelling study
Source: Infect Dis Poverty. 2024 Feb 29;13:21. doi: 10.1186/s40249-024-01189-1 (PMC10902966; doi:10.1186/s40249-024-01189-1)
Supplement: Supplementary file 1 — Additional file 1: Table S1. Summary of epidemiological and clinical features. Table S2. Summary of case number and population of each metropolitan areas among top 12 countries affected by mpox. Table S3. Summary of international airports for each metropolitan area. Table S4. Fitting information for duration of rash. Figure S1. Distribution of the interval from onset to report. (a) The probability density function. (b) The cumulative distribution function. Figure S2. The diagram of simulating the international dissemination of the 2022 multi-country mpox outbreak and evaluating Chinese mainland’s importation risk. In the left panel, we firstly removed all possible imported cases in location i and randomly generated their infectious period according to incubation period. In the right panel, we randomly determine the travel date from their infectiousness period and generate the destination j according to the travel volume. We iterated the entire simulation for 200 times and obtained the distribution of the mpox importations number from location i to location j up to time point t. Figure S3. Validation of the importing risk estimation for 24 countries reporting importation cases more than three times. In each panel, the red ribbon indicates the estimation based on air-travel volumn of 2019 (upper bound estimation) and the blue one indicates that of 2022 (lower bound estimation). Purple dots indicate the observed importations. Table S5. Case Definitions of mpox in Chinese mainland. Table S6. Summary of diagnostic accuracy of confirmation test. Figure S4. Distribution of the number of sexual partners within 1 year among MSM community in Chinese mainland. (a) The cumulative distribution in Chinese mainland. (b) The probability mass distribution of the number of MSM sexual partners in Chinese mainland. In panel a, dots in color of yellow indicated raw data extracted from literature (45, 46). Figure S5. Correlation between the confirmed mpox cases number and active MSM popu [file 40249_2024_1189_MOESM1_ESM.pdf]

Additional file 1 for:

The risk of mpox importation and subsequent outbreak potential in Chinese mainland: a retrospective statistical modelling study

Xiaowei Deng[a†] (0000-0002-9892-2470), Yuyang Tian[a†] (0000-0002-0450-5095), Junyi Zou[a], Juan Yang[a], Kaiyuan Sun[b]*, Hongjie Yu[a]* (0000-0002-6335-5648)

[a]*School of Public Health, Fudan University, Key Laboratory of Public Health Safety, Ministry of Education, Shanghai, China;* [b]*Division of International Epidemiology and Population Studies, Fogarty International Center, National Institutes of Health, Bethesda, MD, USA*

Corresponding authors:
*E-mail: yhj@fudan.edu.cn (Hongjie Yu)
*E-mail: kaiyuan.sun@nih.gov (Kaiyuan Sun)

†These authors contributed equally to this work.
*These authors contributed equally to this work.

Contents

1. Epidemiological and clinical features of mpox 3

2. Metropolitan areas and international airports chosen for analysis from top 12 countries 5

3. Mpox incubation period, duration of rash presentation, and onset to reporting delays 11

4. Mpox onset to reporting delays 12

5. The logic diagram of importation risk simulation 13

6. Validation of the importation risk estimation 14

7. Mpox case definitions 16

8. Border screening 17

9. Outbreak probability 18

10. Correlation between the confirmed mpox cases number and active MSM population size 19

11. Regression models for mpox cases, active MSM population size and air-travel volume 20

12. Country/region ISO Alpha-3 code and the according UN name 21

13. References 22

1. Epidemiological and clinical features of mpox

We summarized 12 published literatures, including 3 from endemic countries (2 from Democratic Republic of the Congo, DRC; 1 from Nigeria), 1 from 2003 USA imported outbreak, and 8 from the recent outbreak in Portugal, Spain, UK, etc. And we searched one relavant literature(1) review, which show similar result with us. The detailed information can be seen in Table S1.

Table S1. Summary of epidemiological and clinical features.

Literature	No. of cases	Location	Period	Confirmation	Sex		HIV infection	Symptoms		
					Male	Female		Fever	Rash	Genital rash
Osadebe et al.(2)	333	Tshuapa Province, DRC [a]	Sept 2009-Feb 2014	PCR	178/333 (53.4%) [b]	154/333 (44.3%) [b]	-	329/329 (100.0%)	316/332 (95.2%)	87/309 (28.2%)
Whitehouse et al.(3)	1057	Tshuapa Province, DRC [a]	2011-2015	PCR	568/1054 (53.9%)	486/1054 (46.1%)	-	1023/1029 (99.4%)	1057/1057 (100.0%)	300/1057 (28.4%)
Yinka-Ogunleye et al.(4)	122 [c]	Nigeria	Sept 22, 2017-Sept 16, 2018	PCR/Antibody detection/Viral isolation	84/122 (68.9%)	38/122 (31.1%)	4 [d]	122/122 (100.0%)	81/92 (88.4%)	44/65 (67.7%)
Huhn et al.(5)	34	USA	2003	PCR/ Viral isolation /Electron microscopy/Antibody detection	18/34 (52.9%)	16/34 (47.1%)	-	29/34 (85.3%)	33/34 (97.1%)	-
Perez Duque et al.(6)	27	Portugal	May 1-23, 2022	PCR	27/27 (100.0%)	0/27 (0.0%)	14/26 (53.8%)	13/26 (50.0%)	14/15 (93.3%)	6/15 (40.0%)
Inigo Martinez et al.(7)	508	Madrid, Spain	May 17-Jun 22, 2022	PCR	503/508 (99.0%)	5/508 (1.0%)	225/508 (44.3%)	324/508 (63.8%)	498/508 (98.0%)	359/508 (72.1%)
Patel et al.(8)	197	London, UK	May 13-July 1, 2022	PCR	197/197 (100.0%)	0/197 (0.0%)	70/197 (35.5%)	122/197 (61.9%)	197/197 (100.0%)	111/197 (56.3%)
Thornhill et al.(9)	528	43 sites in 16 countries	Apr 27-Jun 24, 2022	PCR	527/528 (99.8%) [e]	0/528 (0.0%) [e]	218/528 (41.3%)	330/528 (62.5%)	500/528 (94.7%)	383/528 (72.5%) [f]

Philpott et al.(9)	1195	43 states, Puerto Rico and DC, USA	May 17-July 22, 2022	PCR/Next-Generation sequencing/Viral isolation	1178/1195 (98.7%) [g]	5/1195 (0.4%) [g]	136/334 (41.0%)	596/941 (63.3%)	1004/1004 (100.0%)	333/718 (46.4%)
Tarín-Vicente et al.(10)	181	Madrid and Barcelona, Spain	May 11-Jun 29, 2022	PCR	175/181 (97.0%)	6/181 (3.0%)	72/181 (40.0%)	131/181 (72.0%)	181/181 (100.0%)	100/181 (55.0%)
Català et al.(11)	185	Spain	May 28-July 14, 2022	PCR	185/185 (100.0%)	0/185 (0.0%)	78/185 (42.0%)	100/185 (54%)	185/185 (100.0%)	98/185 (53.0%)
Hoffmann et al.(12)	546	Germany	May 19-Jun 30, 2022	PCR	546/546 (100.0%)	0/546 (0.0%)	256/546 (46.9%)	272/511 (53.2%)	493/546 (90.3%)	267/535 (49.9%)

a. Democratic Republic of the Congo.
b. One missing.
c. Both confirmed and probable case (any suspected case in whom laboratory testing could not be done but who could be epidemiologically linked with a confirmed case).
d. Four of the people who died had HIV with features of AIDS.
e. One nonbinary/trans.
f. Anogenital area.
g. Three transgender men, five transgender women and 4 prefer not to answer.

2. Metropolitan areas and international airports chosen for analysis from top 12 countries

As described in the main text, we assume the travel-associated mpox cases that contribute to the international spread mostly reside in major metropolitan areas with international airport transportation hubs. Consequently, we chose the top 12 countries affected by the 2022 mpox outbreak which account for 90.3% of all mpox cases (as of September 11, 2022). For each country, we focused on the metropolitan areas with most confirmed cases along with major international airports. A full list of selected metropolitan areas, the reported number of mpox cases, along with their corresponding international airports were repored in Table S2 and S3.

Table S2. Summary of case number and population of each metropolitan areas among top 12 countries affected by mpox.

Country	Date of cases reported	Cumulative cases (N)	Metropolitan	Cases within metropolitan (n)	Percent (n/N*100%)	Population
Total	-	58961	-	39114	66.34	268066081
Brazil [a]	2022/9/13	5559(13)	Goiânia	238	4.28	1555626(14)
			São Paulo	2301	41.39	12396372
			Rio de Janeiro	570	10.25	6775561
Canada [a]	2022/10/7	1411(15)	Montreal	449 [b]	31.82	4291732(16)
			Ottawa–Gatineau	41	2.91	1488307
			Toronto	496	35.15	6202225
			Vancouver	165	11.69	2642825
Colombia [a]	2022/10/10	1653(17)	Antioquia	288	17.42	5974788(18)
			Bogota	1148	69.45	7181469
France [a]	2022/9/12	3898(19)	Ile-de-France	2332	61.11	12400000(20)
Germany [a]	2022/10/13	3653(21)	Berlin	1660	45.44	3677472(22)
			North Rhine-Westphalia	796	21.79	17924591
Italy [a]	2022/10/11	856(23)	Lazio	151	17.64	5715190(24)
			Lombardia	350	40.89	9965046
Netherlands [a]	2022/10/7	1219(25)	Noord-Holland & Flevoland	713	58.49	3316712(26)
Peru [a]	2022/9/17	2091(27)	Lima Metropolitan	1648	78.81	10883000(28)
Portugal [a]	2022/9/14	839(29)	Lisboa e Vale do Tejo	650	77.47	2869627(30)
Spain [a]	2022/9/16	7239(31)	Catalonia	2149	29.69	7679410(32)
			Madrid	2500	34.54	6769113
United Kingdom [a]	2022/9/13	3552(33)	London	2335	65.74	8800000(34)
United States [c]	2022/10/7	26991(35)	Atlanta-Sandy Springs-Alpharetta, GA MSA	1437 [d]	5.32	6087762(36)
			Boston-Cambridge-Newton, MA-NH MSA	373 [d]	1.38	4878211
			Chicago-Naperville-Elgin, IL-IN-WI MSA	257	0.95	9515232
			Dallas-Fort Worth-Arlington, TX MSA	989 [e]	3.66	7694138
			Denver-Aurora-Lakewood, CO MSA	221 [d]	0.82	2991231
			Detroit–Warren–Dearborn, MI MSA	124	0.46	4304136
			Houston-The Woodlands-Sugar Land, TX MSA	928 [e]	3.44	7154478
			Los Angeles-Long Beach, CA CSA	2607	9.66	17788274
			Miami-Fort Lauderdale-Pompano Beach, FL MSA	1112	4.12	6173008

			Minneapolis-St. Paul-Bloomington, MN-WI MSA	207 [g]	0.77	3657477
			New York-Newark-Jersey City, NY-NJ-PA MSA	4381 [f, g]	16.23	19068287
			Philadelphia-Camden-Wilmington, PA-NJ-DE-MD MSA	774 [d, g]	2.87	6107906
			Phoenix-Mesa-Chandler, AZ MSA	398	1.47	5059909
			Sacramento-Roseville-Folsom, CA MSA	178 [g]	0.66	2650900
			San Diego-Chula Vista-Carlsbad, CA MSA	404	1.50	3332427
			San Jose-San Francisco-Oakland, CA CSA	1400 [g]	5.19	6668062
			Seattle-Tacoma-Bellevue, WA MSA	550	2.04	4018598
			Tampa-St. Petersburg-Clearwater, FL MSA	213	0.79	3243963
			Washington-Baltimore-Arlington, DC-MD-VA-WV-PA CSA	1581 [d]	5.85	9163016

a. The case number of the metropolitan area was approximately regarded as that reported to the major city (sharing the same name with the metropolitan area).

b. Due to no report at a metro-level in Qubec, Canada, the case number of Montreal was estimated by multiplying the total number of mpox cases in Quebec by the proportion of Toronto's cases in Ontario.

c. The case number of a metropolitan statistical area (MSA) or combined statistical areas (CSA) is the sum of all counties' cases within the MSA/CSA.

d. For these metropolitan areas, case number were not available at county level, but only available at the state level. We assume that the proportion of mpox cases in the metropolitan areas with repect to the state was the same as the proportion of MSM population in the metropolitan area with respect to the state's MSM population. The county-level MSM population estimates for each county were based on Grey et al(37).

e. For Texas, official report was at a level of Public Health Region (PHR). The cases were estimated by multiplying the ratio of MSM of the county to that of the PHR with the PHR's cases.

f. Pike County was unavailable and removed.

g. Reported case number of <5 or <11 was regarded as 5 or 11, respectively.

Table S3. Summary of international airports for each metropolitan area.

Country	Metropolitan	Airport	OAG code
Brazil	Goiânia	Goiânia International Airport	GYN
	Rio de Janeiro	Rio de Janeiro/Galeão International Airport	GIG
		Jacarepaguá Airport	RRJ
		Santos Dumont Airport	SDU
	São Paulo	São Paulo–Congonhas Airport	CGH
		São Paulo/Guarulhos International Airport	GRU
		Jundiaí Airport	QDV
		São José dos Campos Airport	SJK
		Viracopos International Airport	VCP
Canada	Montreal	Montréal–Mirabel International Airport	YMX
		Montreal Saint-Hubert Longueuil Airport	YHU
		Montréal–Trudeau International Airport	YUL
	Ottawa–Gatineau	Gatineau-Ottawa Executive Airport	YND
		Ottawa Macdonald–Cartier International Airport	YOW
	Toronto	Billy Bishop Toronto City Airport	YTZ

		Buttonville Municipal Airport	YKZ
		John C. Munro Hamilton International Airport	YHM
		Region of Waterloo International Airport	YKF
		Toronto Pearson International Airport	YYZ
	Vancouver	Abbotsford International Airport	YXX
		Boundary Bay Airport	YDT
		Vancouver International Airport	YVR
Colombia	Antioquia	José María Córdova International Airport	MDE
		Olaya Herrera Airport	EOH
	Bogota	Bogota FAirport	BOG
France	Ile-de-France	Beauvais–Tillé Airport	BVA
		Châlons Vatry Airport	XCR
		Charles de Gaulle Airport	CDG
		Le Bourget Airport	LBG
		Orly Airport	ORY
Germany	Berlin	Brandenburg Airport	BER
		Schönefeld Airport	SXF
		Tegel Airport	TXL
	North Rhine-Westphalia	Cologne Bonn Airport	CGN
		Dortmund Airport	DTM
		Düsseldorf Airport	DUS
		Münster Osnabrück International Airport	FMO
		Weeze Airport	NRN
Italy	Lazio	Ciampino–G. B. Pastine International Airport	CIA
		Leonardo da Vinci–Fiumicino Airpor	FCO
	Lombardia	Linate Airport	LIN
		Malpensa Airport	MXP
		Orio al Serio International Airport	BGY
		Parma Airport	PMF
Netherlands	Noord-Holland & Flevoland	Amsterdam Schiphol airport	AMS
Peru	Lima	Jorge Chávez International Airport	LIM
Portugal	Lisboa e Vale do Tejo	Lisbon Airport	LIS
Spain	Madrid	Adolfo Suárez Madrid–Barajas Airport	MAD
	Catalonia	Josep Tarradellas Barcelona–El Prat Airport	BCN
		Girona–Costa Brava Airport	GRO
		Lleida–Alguaire Airport	ILD
		Reus Airport	REU
United Kingdom	London	Gatwick Airport	LGW
		Heathrow Airport	LHR
		London City Airport	LCY
		London Southend Airport	SEN
		London Stansted Airport	STN
		Luton Airport	LTN
United States	Atlanta-Sandy Springs-Alpharetta, GA MSA	DeKalb–Peachtree Airport	PDK
		Fulton County Airport (Georgia)	FTY

		Gwinnett County Airport	LZU
		Hartsfield–Jackson Atlanta International Airport	ATL
	Boston-Cambridge-Newton, MA-NH MSA	Beverly Regional Airport	BVY
		Cambridge–Dorchester Airport	CGE
		Hanscom Field	BED
		Lawrence Municipal Airport (Massachusetts)	LWM
		Logan International Airport	BOS
		Manchester–Boston Regional Airport	MHT
		Newton City/County Airport	EWK
		Norwood Memorial Airport	OWD
		Rhode Island T. F. Green International Airport	PVD
		Worcester Regional Airport	ORH
	Chicago-Naperville-Elgin, IL-IN-WI MSA	Chicago Executive Airport	PWK
		Chicago Rockford International Airport	RFD
		DuPage Airport	DPA
		Midway International Airport	MDW
		Milwaukee Mitchell International Airport	MKE
		O'Hare International Airport	ORD
	Dallas-Fort Worth-Arlington, TX MSA	Addison Airport	ADS
		Dallas Fort Worth International Airport	DFW
		Dallas Love Field	DAL
		Fort Worth Alliance Airport	AFW
		Fort Worth Meacham International Airport	FTW
	Denver-Aurora-Lakewood, CO MSA	Centennial Airport	APA
		Aurora Municipal Airport	AUZ
		Rocky Mountain Metropolitan Airport	BJC
		Denver International Airport	DEN
	Detroit–Warren–Dearborn, MI MSA	Ann Arbor Municipal Airport	ARB
		Coleman A. Young International Airport	DET
		Detroit Metropolitan Airport	DTW
		Bishop International Airport	FNT
		St. Clair County International Airport	PHN
		Oakland County International Airport	PTK
		Willow Run Airport	YIP
	Houston-The Woodlands-Sugar Land, TX MSA	David Wayne Hooks Memorial Airport	DWH
		Ellington Airport (Texas)	EFD
		William P. Hobby Airport	HOU
		George Bush Intercontinental Airport	IAH
		West Houston Airport	IWS
		Sugar Land Regional Airport	SGR
	Los Angeles-Long Beach, CA CSA	Hollywood Burbank Airport	BUR
		Los Angeles International Airport	LAX
		Long Beach Airport	LGB
		Ontario International Airport	ONT
		San Bernardino International Airport	SBD
		John Wayne Airport	SNA
		Van Nuys Airport	VNY
		Boca Raton Airport	BCT

	Miami-Fort Lauderdale-Pompano Beach, FL MSA	Fort Lauderdale–Hollywood International Airport	FLL
		Fort Lauderdale Executive Airport	FXE
		Homestead Air Reserve Base	HST
		Miami International Airport	MIA
		Miami-Opa Locka Executive Airport	OPF
		Palm Beach International Airport	PBI
		Pompano Beach Airpark	PPM
		Miami Executive Airport	TMB
		Dade-Collier Training and Transition Airport	TNT
	Minneapolis-St. Paul-Bloomington, MN-WI MSA	Flying Cloud Airport	FCM
		Crystal Airport (Minnesota)	MIC
		Minneapolis–Saint Paul International Airport	MSP
		St. Paul Downtown Airport	STP
	New York-Newark-Jersey City, NY-NJ-PA MSA	Newark Liberty International Airport[a]	EWR
		Westchester County Airport	HPN
		Tweed New Haven Airport	HVN
		Long Island MacArthur Airport	ISP
		John F. Kennedy International Airport	JFK
		LaGuardia Airport	LGA
		New York Skyports Seaplane Base	NYS
		Stewart International Airport	SWF
		Trenton–Mercer Airport[a]	TTN
	Philadelphia-Camden-Wilmington, PA-NJ-DE-MD MSA	Lehigh Valley International Airport	ABE
		Atlantic City International Airport	ACY
		Wilmington Airport (Delaware)	ILG
		Philadelphia International Airport	PHL
		Northeast Philadelphia Airport	PNE
		Reading Regional Airport	RDG
	Phoenix-Mesa-Chandler, AZ MSA	Phoenix–Mesa Gateway Airport	AZA
		Phoenix Deer Valley Airport	DVT
		Phoenix Goodyear Airport	GYR
		Falcon Field (Arizona)	MSC
		Phoenix Sky Harbor International Airport	PHX
		Scottsdale Airport	SCF
	Sacramento-Roseville-Folsom, CA MSA	Sacramento McClellan Airport	MCC
		Sacramento Mather Airport	MHR
		Sacramento Executive Airport	SAC
		Stockton Metropolitan Airport	SCK
		Sacramento International Airport	SMF
	San Diego-Chula Vista-Carlsbad, CA MSA	McClellan–Palomar Airport	CLD
		Montgomery-Gibbs Executive Airport	MYF
		San Diego International Airport	SAN
		Brown Field Municipal Airport	SDM
		Gillespie Field	SEE
	San Jose-San Francisco-Oakland, CA CSA	Oakland International Airport	OAK
		Reid–Hillview Airport	RHV
		San Francisco International Airport	SFO
		San Jose International Airport	SJC

	Seattle-Tacoma-Bellevue, WA MSA	Boeing Field	BFI
		Kenmore Air Harbor Seaplane Base	LKE
		Paine Field	PAE
		Renton Municipal Airport	RNT
		Seattle–Tacoma International Airport	SEA
		Tacoma Narrows Airport	TIW
	Tampa-St. Petersburg-Clearwater, FL MSA	Clearwater Air Park Airport	CLW
		St. Pete–Clearwater International Airport	PIE
		Albert Whitted Airport	SPG
		Tampa International Airport	TPA
		Peter O. Knight Airport	TPF
	Washington-Baltimore-Arlington, DC-MD-VA-WV-PA CSA	Baltimore/Washington International Airport	BWI
		Ronald Reagan Washington National Airport	DCA
		Washington Dulles International Airport	IAD
		Martin State Airport	MTN

[a] These airports serve both New York-Newark-Jersey City, NY-NJ-PA MSA and Philadelphia-Camden-Wilmington, PA-NJ-DE-MD MSA. Here for simplicity, we assume these airports only serve NY-NJ-PA MSA according to their locations.

3. Mpox incubation period, duration of rash presentation, and onset to reporting delays

Based on the estimation by Miura et al(38), the incubation period of mpox infection in the Netherlands had a median of 8.5 days with 5th–95th percentiles of 4.2–17.3 days. The distribution of the incubation period is best fitted by a lognormal distribution. We assume the duration from infection to symptom onset follows the same distribution.

The duration of rash is estimated using the empirical data from Huhn et al(5). We assume the range as the 2.5th and 97.5th percentiles. Lognormal, Gamma, and Weibull distribution are fitted using nonlinear least squares method. The distribution with the minimum sum of square residual is chosen as the distribution for the duration of rash (Table S4).

Table S4. Fitting information for duration of rash.

Distribution	Parameter 1	Parameter 2	Residual (sum of square)
Gamma	15.32373478	1.248809497	0.000572
Lognormal	2.486530301	0.29177409	0.000313
Weibull	6.140548705	12.73805504	0.000625

4. Mpox onset to reporting delays

We downloaded the empirical data of the time interval from onset to reporting from the WHO situation dashboard(39). Then, we fit the data using zero-inflated passion (hereafter ZIP for short) and zero-inflated negative binomial (hereafter ZINBI for short) through a maximum likelihood method. The fitting of ZINBI is better, which can be seen in Figure S1.

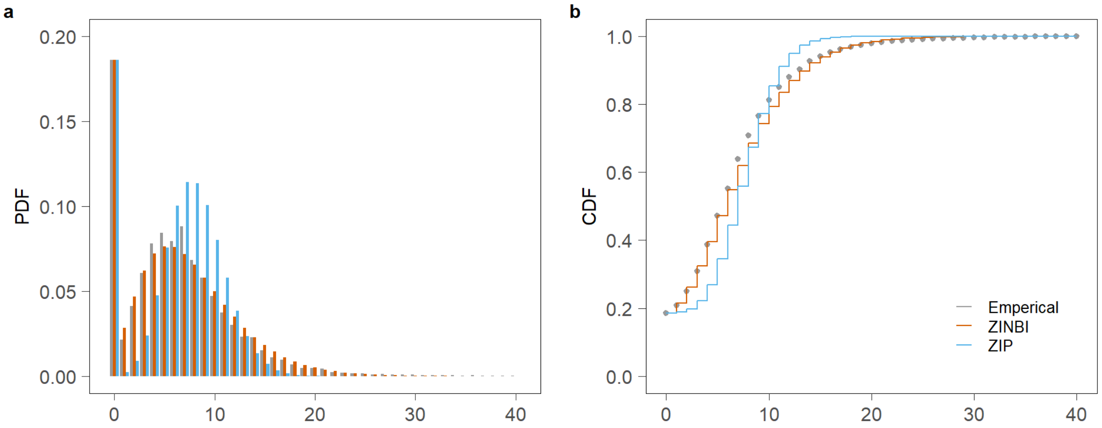

Figure S1. Distribution of the interval from onset to report. (a) The probability density function. (b) The cumulative distribution function.

5. The logic diagram of importation risk simulation

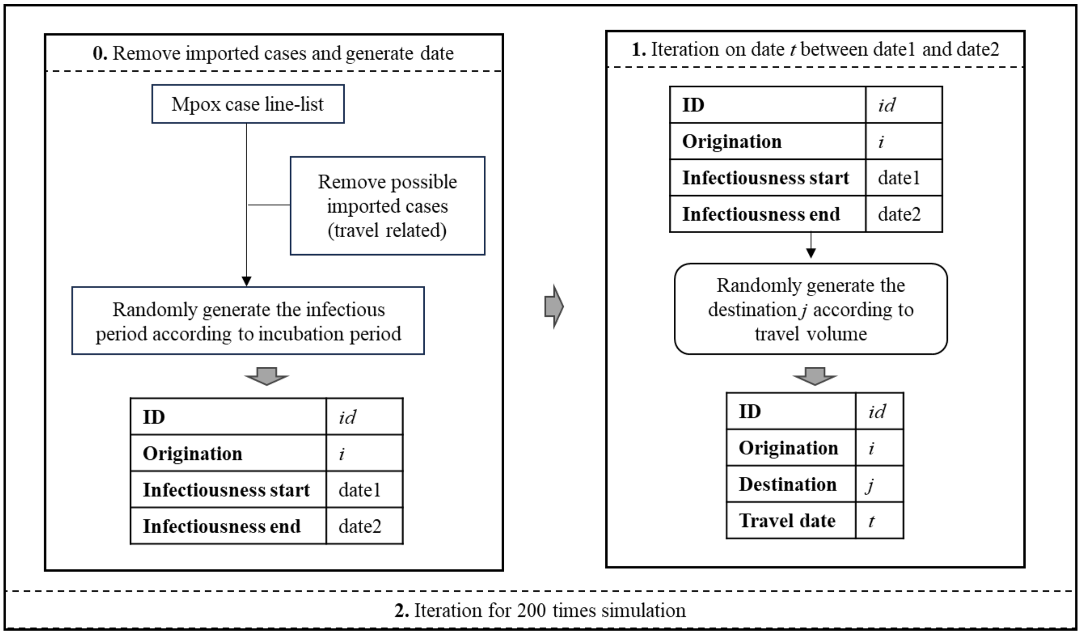

Figure S2. The diagram of simulating the international dissemination of the 2022 multi-country mpox outbreak and evaluating Chinese mainland's importation risk. In the left panel, we firstly removed all possible imported cases in location *i* and randomly generated their infectious period according to incubation period. In the right panel, we randomly determine the travel date from their infectiousness period and generate the destination *j* according to the travel volume. We iterated the entire simulation for 200 times and obtained the distribution of the mpox importations number from location *i* to location *j* up to time point *t*.

6. Validation of the importation risk estimation

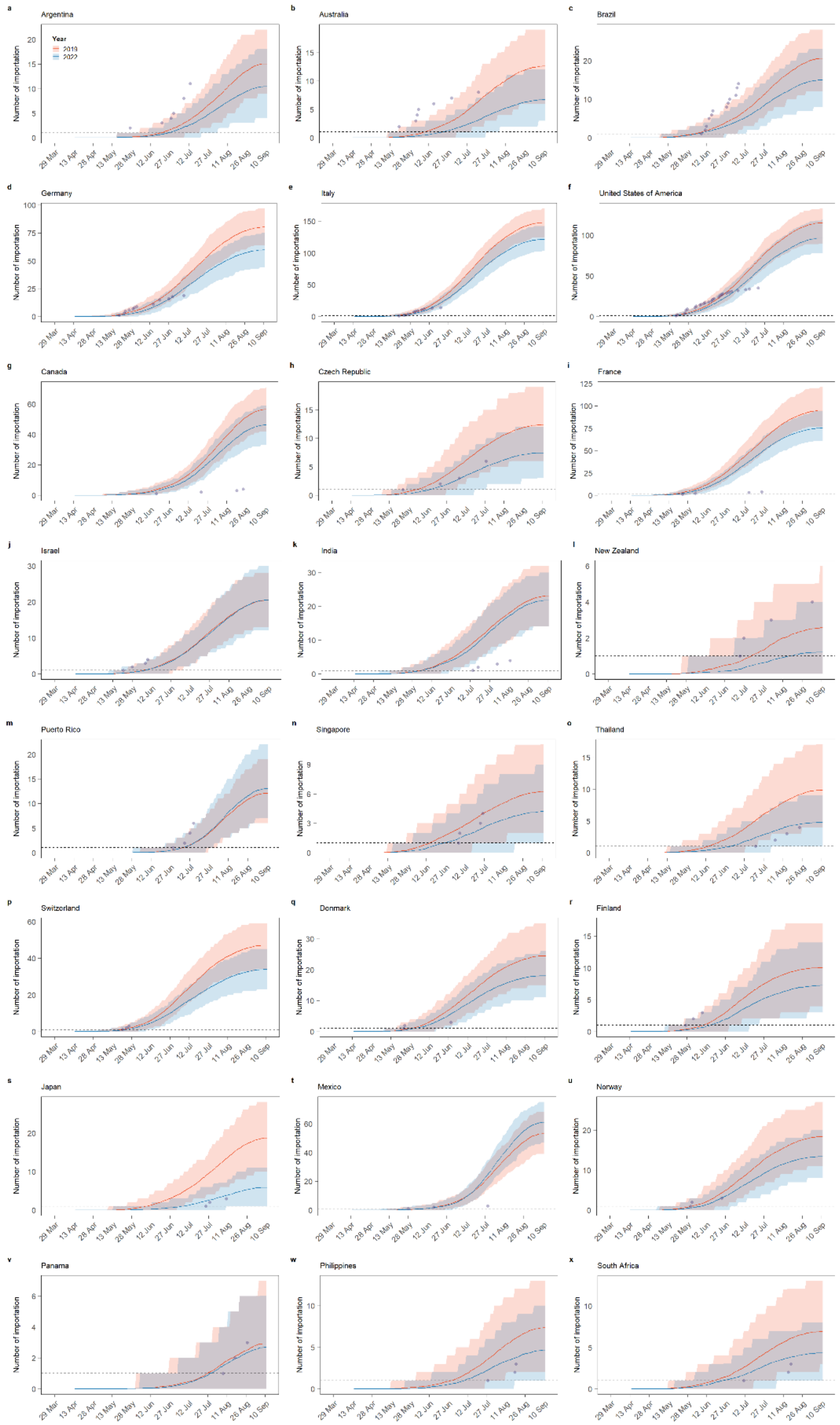

Figure S3. Validation of the importing risk estimation for 24 countries reporting importation cases more than three times. In each panel, the red ribbon indicates the estimation based on air-travel volumn of 2019 (upper bound

estimation) and the blue one indicates that of 2022 (lower bound estimation). Purple dots indicate the observed importations.

7. Mpox case definitions

Table S5. Case Definitions of mpox in Chinese mainland.

Class	Definitions
Suspect Case	With symptoms of fever, swollen lymph nodes and skin or mucosal rash and with any of the following epidemiological history:<br>Within 21 days of illness onset:<br>● Having overseas travel history in areas with mpox case reports.<br>● Having close contact with a confirmed case of mpox.<br>● Exposed to blood, body fluids and secretions of mpox-infected animals such as rodents and non-human primates.
Confirmed Case	Meeting the definition of suspected cases, laboratory tests, mpox virus nucleic acid positive or virus isolation positive

8. Border screening

In Scenario 1 corresponding to the Chinese mainland's quarantine policy till June 28, 2022, we consider all international travelers arriving at Chinese mainland need to undergo an entry quarantine, but with varying quarantine duration through the year of 2022 due to policy adjustment over time(40). In Scenario 2, only individuals with known exposure to mpox need to undergo medical observations for mpox related symptoms. During the medical observation, exposed individuals need to abstain from sexual activity, along with other precautious measures. No mandatory isolation is required unless exposed individuals start developing mpox related symptoms during the period of medical observation. Both confirmed and suspected mpox cases need to be isolated at the designated medical facilities and we assume they could not further transmit mpox to others in our analysis.

We simulated 100 mpox infected entry personals, among whom 90% would develop rash following a Bernoulli process. We assigned each individual's the incubation period according to a lognormal distribution(38) with mean of 6.48 days and the standard deviation of 28.2 days. We assume the duration of rash following a lognormal (Table S4) with mean of 2.49 days and the standard deviation of 0.29 days. Then, we randomly assigned the border entry date of each infected indivudal with unifom likelihood within the time window of his/her incubation. For Scenario 1, where a 7-day mandatory quarantine is required, mpox PCR test would be performed once rash onset. On the contrary, for Scenario 2, only people reporting the epidemiological link would seek tests once rash onset. For each scenario, we simulated the performance of PCR test following a Bernoulli process with 95% sensitivity based on three published articles(41–43). We repeated the entire simulation for 200 times to obtain the distribution.

The probability of mpox infection detection was presented as a function of the COVID-19 quarantine duration and the proportion of self-reporting epidemiological links for Scenario 1. As for Scenario 2, the effectiveness of the border screening policy was explored as a function of the proportion of self-reporting epidemiological links and the duration of the medical observation among imported mpox infections.

The sensitivity of PCR confirmation of mpox is based on the summary of three articles described in Table S6.

Table S6. Summary of diagnostic accuracy of confirmation test.

**Ref**	**Davi et al.(41)**		**Li et al. 2010.(42)**			**Li et al. 2006.(43)**
Method	RPA[a]	rt-PCR	rt-PCR			rt-PCR
Target	G2R	G2R-G	G2R-WA	G2R-G	C3L	B6R
Purpose	both clades of MPXV		West African clade	both clades of MPXV	Congo Basin clade	MPXV
Specimen	Whole blood/serum		liver and skin tissues, nasal and oral swabs, lesions and human mpox clinical samples			Samples of skin, slides, and swabs.
Positive	25 monkeys and 20 humans		4 strains, 10 animals and 3 humans	11 strains, 20 animals and 7 humans	7 strains, 10 animals and 4 humans	1 MPXV DNA samples and 13 human clinical samples
Negative	23 monkeys and 27 humans		14 strains, 3 other DNA, 10 animals and 4 humans	7 strains and 3 other DNA	11 strains, 3 other DNA, 10 animals and 3 humans	9 *orthopoxviruses* DNA samples, 15 bacteria DNA samples and 2 VZV[b] clinical samples
Result						
Sensitivity	43/45 (95.6%)	45/45 (100.0%)	17/17 (100.0%)	38/38 (100.0%)	21/21 (100.0%)	14/14 (100.0%)
Specificity	50/50 (100.0%)	50/50 (100.0%)	31/31 (100.0%)	10/10 (100.0%)	27/27 (100.0%)	26/26 (100.0%)

a. Recombinase polymerase amplification.
b. Varicella zoster virus.

9. Outbreak probability

Following Hartfield et al.'s analytical solution of outbreak probability T_0 of a single seeding case as a function of the basic reproduction number R_0 and dispersion parameter k.

$$T_0 = \frac{1}{\log R_0}(0.334 + \frac{0.689}{k} + \frac{0.408}{R_0} - \frac{0.507}{kR_0} - \frac{0.356}{{R_0}^2} + \frac{0.467}{k{R_0}^2})$$

A reasonable guess of the mpox's basic reproduction number among MSM community in Chinese mainland can be approximated by what's being observed in other non-endemic countries with substential outbreak size ($R_0 = 1.8$ base a recent study by Kwok et al(44)).

We use the distribution of the number of sexual partners among MSM in Chinese mainland to approximate the dispersion of mpox transmission k. Figure S3 displays the observed distribution of the number of sexual partners among MSM in Chinese mainland(45, 46). The number of sexual partners ranges from 1 to 53, which can be characterized by a negative binomial distribution with mean $\mu = 3.04$ and variance $\mu(1 + \mu/k) = 13.58$, where $k = 0.88$ is the dispersion parameter estimated by the distribution fitting.

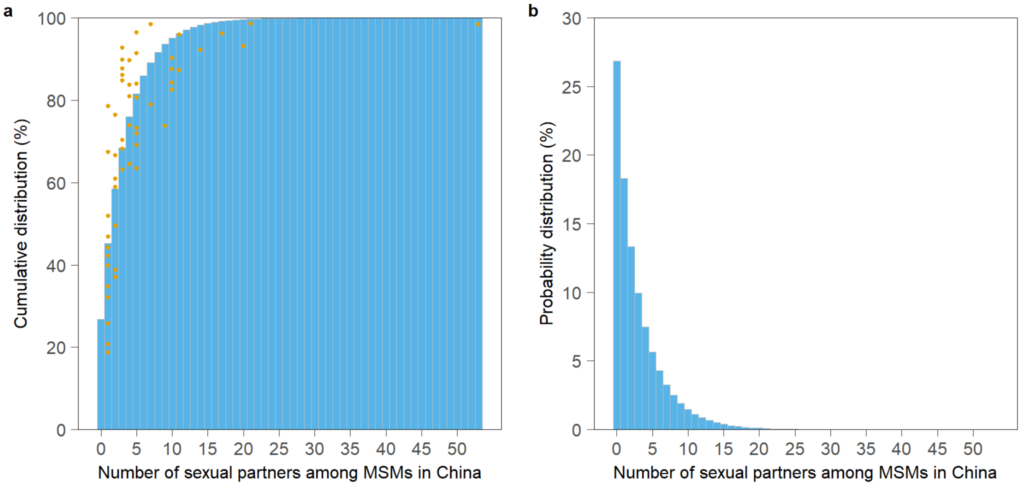

Figure S4. Distribution of the number of sexual partners within 1 year among MSM community in Chinese mainland. (a) The cumulative distribution in Chinese mainland. (b) The probability mass distribution of the number of MSM sexual partners in Chinese mainland. In panel a, dots in color of yellow indicated raw data extracted from literature(45, 46).

10. Correlation between the confirmed mpox cases number and active MSM population size

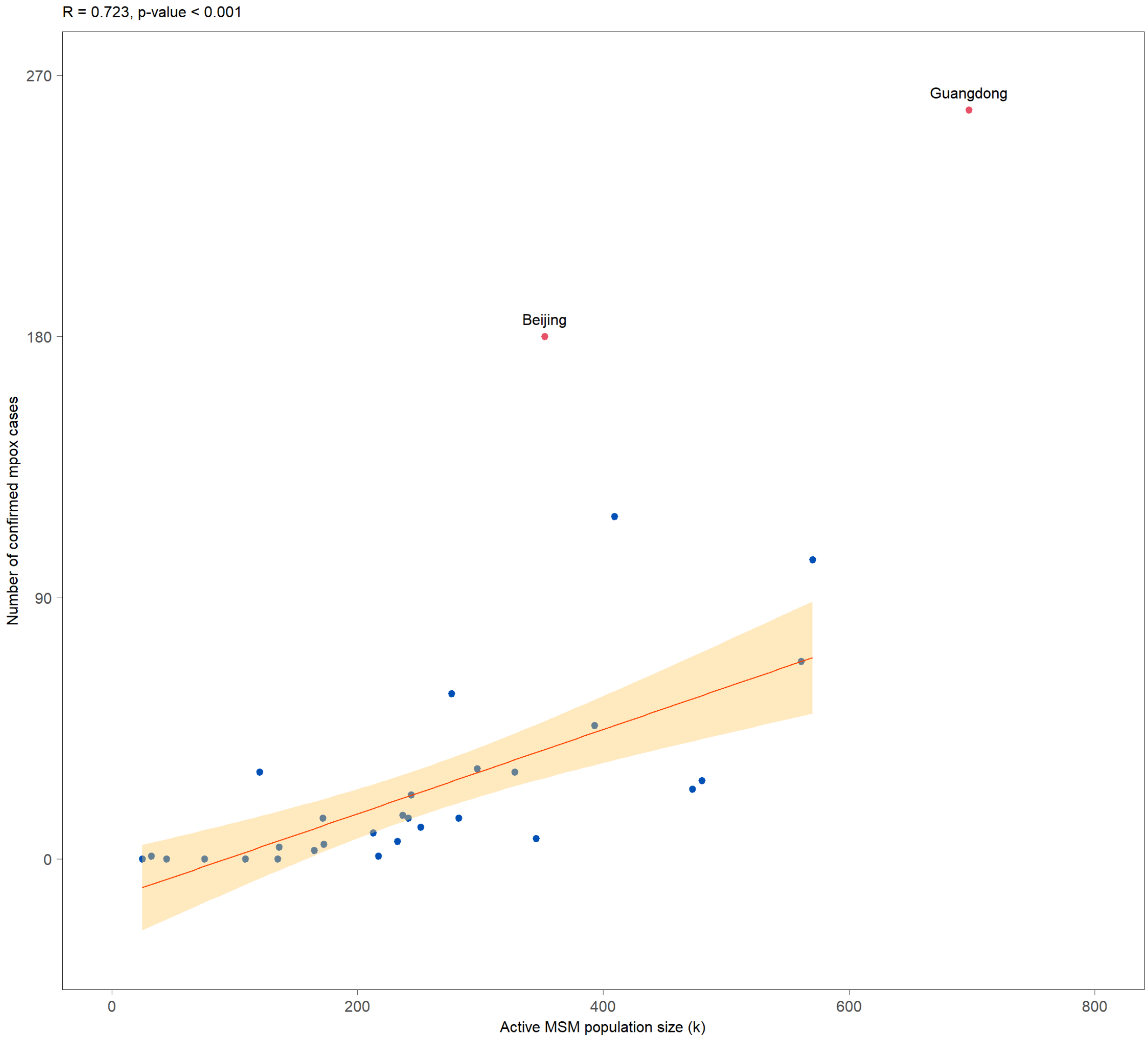

Figure S5. Correlation between the confirmed mpox cases number and active MSM population size of 29 provinces in Chinese mainland. The orange ribbon indicates the estimation based on the active MSM population size(47). The blue dots indicate different provinces in Chinese mainland and the two red dots present outliers in the analysis.

11. Regression models for mpox cases, active MSM population size and air-travel volume

Table S7. R^2 for different regression combination

Var	31 provinces	29 provinces [a]
active MSM population size	0.4919	0.5046
2019 air-travel volume	0.3498	0.1603
active MSM population size + 2019 air-travel volume	0.6152	0.5539
active MSM population size + 2019 air-travel volume + active MSM population size * 2019 air-travel volume	0.84	0.5964

a. Excluded Beijing and Guangdong province.

For combination including active MSM population size, 2019 air-travel volume and their interaction term (R^2=0.84), the formula is described as:

$$\mu_i = \alpha + \beta_1 x_1 + \beta_2 x_2 + \beta_3 x_1 x_2,$$

where μ_i is the confirmed mpox cases number, x_1 is the active MSM population size (100 thousand), x_2 is the 2019 air-travel volume (100 thousand), $x_1 x_2$ is the interaction term, α is the constant term and β_i is the coefficient.

12. Country/region ISO Alpha-3 code and the according UN name

Table S8. Country/region ISO Alpha-3 code and the according UN name.

ISO Alpha-3	Name by UN
BRA	Brazil
CAN	Canada
COL	Colombia
DEU	Germany
ESP	Spain
FRA	France
GBR	United Kingdom
ITA	Italy
NLD	Netherlands
PER	Peru
PRT	Portugal
USA	United States of America

13. References

1. Bragazzi NL, Kong JD, Mahroum N, Tsigalou C, Khamisy-Farah R, Converti M, et al. Epidemiological trends and clinical features of the ongoing monkeypox epidemic: A preliminary pooled data analysis and literature review. J Med Virol. 2022. doi: 10.1002/jmv.27931.
2. Osadebe L, Hughes CM, Shongo Lushima R, Kabamba J, Nguete B, Malekani J, et al. Enhancing case definitions for surveillance of human monkeypox in the Democratic Republic of Congo. PLoS Negl Trop Dis. 2017; 11(9): e0005857. doi: 10.1371/journal.pntd.0005857.
3. Whitehouse ER, Bonwitt J, Hughes CM, Lushima RS, Likafi T, Nguete B, et al. Clinical and Epidemiological Findings from Enhanced Monkeypox Surveillance in Tshuapa Province, Democratic Republic of the Congo During 2011-2015. J Infect Dis. 2021; 223(11): 1870-1878. doi: 10.1093/infdis/jiab133.
4. Yinka-Ogunleye A, Aruna O, Dalhat M, Ogoina D, McCollum A, Disu Y, et al. Outbreak of human monkeypox in Nigeria in 2017-18: a clinical and epidemiological report. Lancet Infect Dis. 2019; 19(8): 872-879. doi: 10.1016/S1473-3099(19)30294-4.
5. Huhn GD, Bauer AM, Yorita K, Graham MB, Sejvar J, Likos A, et al. Clinical characteristics of human monkeypox, and risk factors for severe disease. Clin Infect Dis. 2005; 41(12): 1742-1751. doi: 10.1086/498115.
6. Perez Duque M, Ribeiro S, Martins JV, Casaca P, Leite PP, Tavares M, et al. Ongoing monkeypox virus outbreak, Portugal, 29 April to 23 May 2022. Euro Surveill. 2022; 27(22). doi: 10.2807/1560-7917.ES.2022.27.22.2200424.
7. Inigo Martinez J, Gil Montalban E, Jimenez Bueno S, Martin Martinez F, Nieto Julia A, Sanchez Diaz J, et al. Monkeypox outbreak predominantly affecting men who have sex with men, Madrid, Spain, 26 April to 16 June 2022. Euro Surveill. 2022; 27(27). doi: 10.2807/1560-7917.ES.2022.27.27.2200471.
8. Patel A, Bilinska J, Tam JCH, Da Silva Fontoura D, Mason CY, Daunt A, et al. Clinical features and novel presentations of human monkeypox in a central London centre during the 2022 outbreak: descriptive case series. Bmj. 2022; 378: e072410. doi: 10.1136/bmj-2022-072410.
9. Thornhill JP, Barkati S, Walmsley S, Rockstroh J, Antinori A, Harrison LB, et al. Monkeypox Virus Infection in Humans across 16 Countries - April-June 2022. N Engl J Med. 2022. doi: 10.1056/NEJMoa2207323.
10. Tarín-Vicente EJ, Alemany A, Agud-Dios M, Ubals M, Suñer C, Antón A, et al. Clinical presentation and virological assessment of confirmed human monkeypox virus cases in Spain: a prospective observational cohort study. Lancet. 2022. doi: 10.1016/s0140-6736(22)01436-2.
11. Català A, Clavo Escribano P, Riera J, Martín-Ezquerra G, Fernandez-Gonzalez P, Revelles Peñas L, et al. Monkeypox outbreak in Spain: clinical and epidemiological findings in a prospective cross-sectional study of 185 cases. Br J Dermatol. 2022. doi: 10.1111/bjd.21790.
12. Hoffmann C, Jessen H, Wyen C, Grunwald S, Noe S, Teichmann J, et al. Clinical

characteristics of monkeypox virus infections among men with and without HIV: A large outbreak cohort in Germany. HIV Med. 2022. doi: 10.1111/hiv.13378.
13. Ministério da Saúde. Boletim Epidemiológico de Monkeypox nº 8 (COE). https://www.gov.br/saude/pt-br/centrais-de-conteudo/publicacoes/boletins/epidemiologicos/variola-dos-macacos/boletim-epidemiologico-de-monkeypox-no-11-coe/view. Accessed Oct 7. (in Portuguese)
14. Wikipedia. List of metropolitan areas in Brazil. https://en.wikipedia.org/wiki/List_of_metropolitan_areas_in_Brazil. Accessed Sep 20.
15. Government du Canada. Variole simienne : Mise à jour sur l'éclosion. https://www.canada.ca/fr/sante-publique/services/maladies/variole-singe/mise-jour-eclosion.html#a1. Accessed Oct 7. (in French)
16. Wikipedia. List of census metropolitan areas and agglomerations in Canada. https://en.wikipedia.org/wiki/List_of_census_metropolitan_areas_and_agglomerations_in_Canada. Accessed Sep 20.
17. Ministerio de Salud y Protección Social. VIRUELA SÍMICA (MONKEYPOX). https://app.powerbi.com/view?r=eyJrIjoiNjQzZGQ0ZjAtODRhMi00MzFhLWFmODctOWMxNGVkNGFjMDhkIiwidCI6ImE2MmQ2YzdiLTlmNTktNDQ2OS05MzU5LTM1MzcxNDc1OTRiYiIsImMiOjR9. Accessed Oct 10. (in Spanish)
18. Departamento Administrativo Nacional de Estadística. ¿CUÁNTOS SOMOS? https://sitios.dane.gov.co/cnpv/#!/. Accessed Sep 20. (in Spanish)
19. Santé Publique France. Cas de variole du singe : point de situation au 12 septembre 2022. https://www.santepubliquefrance.fr/les-actualites/2022/cas-de-variole-du-singe-point-de-situation-au-12-septembre-2022. Accessed Sep 20. (in French)
20. Wikipedia. Île-de-France. https://fr.wikipedia.org/wiki/%C3%8Ele-de-France#Population_et_soci%C3%A9t%C3%A9. Accessed Sep 20.
21. Robert Koch-Institut. Abfrage erstellen. https://survstat.rki.de/Content/Query/Create.aspx. Accessed 13 Oct 2022.
22. Statistisches Bundesamt. Population by nationaly and federal states. https://www.destatis.de/EN/Themes/Society-Environment/Population/Current-Population/Tables/population-by-laender.html. Accessed Sep 20.
23. Ministero della Salute. Vaiolo delle scimmie. https://www.salute.gov.it/portale/malattieInfettive/dettaglioSchedeMalattieInfettive.jsp?lingua=italiano&id=254&area=Malattie%20infettive&menu=indiceAZ&tab=1. Accessed Oct 11. (in Italian)
24. I.Stat. http://dati.istat.it/?lang=en. Accessed Sep 20.
25. Ministry of Health, Welfare and Sport. Monkeypox. https://www.rivm.nl/en/monkeypox. Accessed Oct 7.
26. StatLine. Population dynamics; birth, death and migration per region. https://opendata.cbs.nl/statline/#/CBS/en/dataset/37259eng/table?ts=1661681544027. Accessed Sep 20.
27. Minsa Peru. https://twitter.com/Minsa_Peru/status/1570915777406201857. Accessed Sep 20.
28. Macrotrends. Lima, Peru Metro Area Population 1950-2022. https://www.macrotrends.net/cities/22078/lima/population. Accessed Sep 20.

29. Serviço Nacional de Saúde. Informação semanal sobre Infeção Humana por vírus Monkeypox – Nº 008. https://www.dgs.pt/em-destaque/informacao-semanal-sobre-infecao-humana-por-virus-monkeypox-n-011-pdf.aspx. Accessed Sep 20. (in Portuguese)
30. Instituto Nacional de Estatística. Estimativas de População Residente. https://www.ine.pt/xportal/xmain?xpid=INE&xpgid=ine_destaques&DESTAQUESdest_boui=540837471&DESTAQUESmodo=2. Accessed Sep 20. (in Portuguese)
31. Ministerio de Sanidad. Informe_de_situacion_MPX_20221011. https://www.sanidad.gob.es/profesionales/saludPublica/ccayes/alertasActual/alertaMonkeypox/docs/Informe_de_situacion_MPX_20221011.pdf. Accessed 20 Sep 2022. (in Spanish)
32. Instituto Nacional de Estadística. Population figures. Latest data. https://www.ine.es/dyngs/INEbase/en/operacion.htm?c=Estadistica_C&cid=1254736176951&menu=ultiDatos&idp=1254735572981. Accessed Sep 20. (in Spanish)
33. UK Government. Monkeypox outbreak: epidemiological overview, 23 August 2022. https://www.gov.uk/government/publications/monkeypox-outbreak-epidemiological-overview/monkeypox-outbreak-epidemiological-overview-13-september-2022. Accessed Sep 20.
34. Offical of National Statistics. Population and household estimates, England and Wales: Census 2021. https://www.ons.gov.uk/peoplepopulationandcommunity/populationandmigration/populationestimates/bulletins/populationandhouseholdestimatesenglandandwales/census2021. Accessed Sep 20.
35. Centers for Disease Control and Prevention. 2022 U.S. Map & Case Count. https://www.cdc.gov/poxvirus/monkeypox/response/2022/us-map.html. Accessed 7 Oct 2022.
36. Wikipedia. Metropolitan statistical area. https://en.wikipedia.org/wiki/Metropolitan_statistical_area. Accessed Sep 20.
37. Grey JA, Bernstein KT, Sullivan PS, Purcell DW, Chesson HW, Gift TL, et al. Estimating the Population Sizes of Men Who Have Sex With Men in US States and Counties Using Data From the American Community Survey. JMIR Public Health Surveill. 2016; 2(1): e14. doi: 10.2196/publichealth.5365.
38. Miura F, van Ewijk CE, Backer JA, Xiridou M, Franz E, Op de Coul E, et al. Estimated incubation period for monkeypox cases confirmed in the Netherlands, May 2022. Euro Surveill. 2022; 27(24). doi: 10.2807/1560-7917.ES.2022.27.24.2200448.
39. World Health Organization. 2022 Monkeypox Outbreak: Global Trends. https://worldhealthorg.shinyapps.io/mpx_global/. Accessed 19 June 2023.
40. The Central People's Government of the People's Republic of China. Interpretation of the "Prevention and Control Strategy of COVID-19 (9th edn)". http://www.gov.cn/zhengce/2022-06/28/content_5698169.htm. Accessed 20 Sep 2022. (in Chinese)
41. Davi SD, Kissenkotter J, Faye M, Bohlken-Fascher S, Stahl-Hennig C, Faye O, et al. Recombinase polymerase amplification assay for rapid detection of Monkeypox virus. Diagn Microbiol Infect Dis. 2019; 95(1): 41-45. doi: 10.1016/j.diagmicrobio.2019.03.015.
42. Li Y, Zhao H, Wilkins K, Hughes C, Damon IK. Real-time PCR assays for the specific detection

of monkeypox virus West African and Congo Basin strain DNA. J Virol Methods. 2010; 169(1): 223-227. doi: 10.1016/j.jviromet.2010.07.012.
43. Li Y, Olson VA, Laue T, Laker MT, Damon IK. Detection of monkeypox virus with real-time PCR assays. J Clin Virol. 2006; 36(3): 194-203. doi: 10.1016/j.jcv.2006.03.012.
44. Kwok KO, Wei WI, Tang A, Shan Wong SY, Tang JW. Estimation of local transmissibility in the early phase of monkeypox epidemic in 2022. Clin Microbiol Infect. 2022. doi: 10.1016/j.cmi.2022.06.025.
45. Zhang L, Chow EP, Wilson DP. Distributions and trends in sexual behaviors and HIV incidence among men who have sex with men in China. BMC Public Health. 2012; 12: 546. doi: 10.1186/1471-2458-12-546.
46. Zhang L, Fung Chow EP, Wilson DP. Men who have sex with men in China have relatively low numbers of sexual partners. Infect Dis Rep. 2011; 3(1): e10. doi: 10.4081/idr.2011.e10.
47. Hu M, Xu C, Wang J. Spatiotemporal Analysis of Men Who Have Sex With Men in Mainland China: Social App Capture-Recapture Method. JMIR Mhealth Uhealth. 2020; 8(1): e14800. doi: 10.2196/14800.
